# Nouvelles Recherches sur le Rheumatisme Articulaire Aigu, &c.—New Researches Respecting Acute Articular Rheumatism in General, and Especially on the Law of Coincidence between That Disease and Pericarditis, &c.

**Published:** 1836-07-01

**Authors:** 


### NOUVELLES RECHERCHES SUR LE RHEUMATISME ARTICULAIRE AIGU EN GÉNÉRAL, ET SPECIALEMENT SUR LA LOI DE COÏNCIDENCE DE LA PERICARDITE ET DE L'ENDOCARDITE AVEC CETTE MALADIE, AINSI QUE SUR L'EFFICACITÉ DE LA FORMULE DES EMISSIONS SANGUINES COUP SUR COUP DANS SON TRAITEMENT. Par *J. Bouillaud.* Paris, 1836.

M. BOUILLAUD is well known to the profession as not only an enlightened and ingenious, but also a most indefatigable physician. His " Clinical Treatise on Diseases of the Heart" was reviewed by us in our last Number; and, from the analysis there given of it, our readers must have perceived that it is a work of no ordinary merit. In it the author dwells at great length upon a fact, with which it behoves both the practitioner and the pathologist to be well acquainted—namely, that inflammatory affections of the heart, as well as of the pericardium, are very generally connected with, or consequent upon, acute articular rheumatism. M. Bouillaud, while urging with earnestness the verity of this connexion, uses, it is true, expressions at which the British reader can scarcely refrain from smiling—claiming for himself the honor of having been the first to discover it, and wondering how it happened to escape the notice of the many observant physicians who had previously treated of rheumatism.

" I venture," says he (in the preface to the work before us), "to announce as a discovery worthy of some attention, the almost constant coincidence with violent acute articular rheumatism of inflammation of the lining membrane of the heart *(endocarditis),* or of *pericarditis,* or of both conjoined—*endo-pericarditis.* This fact, which the observations of each succeeding day only serve to confirm, is one of such importance, that it constitutes, to a certain extent, a real revolution in the history of acute articular rheumatism. It is not surprising that there have been found some to contradict it: such is the fate of all new and important truths. But I hesitate not to predict that, ere long, men will fail to comprehend how a fact of such frequent occurrence could have eluded the attention of the many labourers who have preceded us in the field of observation."

The state of the profession in France may, perhaps, justify in some measure, though certainly not altogether, the claims to discovery here put forward by M. Bouillaud; but one cannot help feeling surprised that so well-informed a physician should be ignorant, that the frequent occurrence of pericarditis and other affections of the heart, in connexion with acute rheumatism, has been universally recognized by British practitioners ever since their attention was first directed to it by Sir David Dundas. In none of our later systematic works on medicine is the subject passed unnoticed; in our "Cyclopædia of Medicine," it is ably and minutely discussed; and the pages of our periodical press afford incontestable proof of the importance which we attach to it. Yet M. Bouillaud refers only to the work of Dr. Hope, whose brief remarks on the subject he quotes, declaring, at the same time, that he was completely ignorant of them until after he himself had made the discovery. He animadverts, with reason, upon an assertion of Dr. Hope—that the patient almost always falls a victim to the immediate effects of the disease, when violent, and that, even then, it is extremely obscure in its symptoms, as well as insidious in its progress. Such a statement we hear very frequently made, and yet nothing, we feel assured from experience, can be more unfounded. It may be difficult, if not impossible, to diagnose positively, in every case where it exists, mere effusion of serum into the pericardium; but to the expert physician, even the slighter degrees of inflammation of that sac are, we maintain, always discoverable. As to active inflammation of the pericardium, that is attended with symptoms so violent, and at the same time so characteristic, that we wonder how any man, possessing eyes and ears, can ever fail to satisfy himself of its existence.

Yet there is one point of view in which M. B. may, not altogether without reason, be regarded as a discoverer. It has been the general, if not the universal custom, to look upon pericarditis or endocarditis (this very appropriate name is of M. B.'s own coinage) as merely an *accidental occurrence* during the progress of rheumatism, or, at most, nothing more than the result of a *metastasis*, or *transferred* morbid action. M. B. on the contrary, has satisfied himself that both these affections "constitute, in some measure, *natural elements of the disease,* which, regarded in a juster and more comprehensive point of view, consists in inflammation of the fibro-serous tissues in general." This observation merits, in our opinion, the greatest attention, for, by representing inflammatory affections of the heart and pericardium as constituent elements of rheumatism, and not merely as accessary and contingent events, it must lead practitioners to be more suspicious of their existence, and, by detecting them earlier, to treat them more successfully. M. B. avers that endo-pericarditis exists in fully one-half of the individuals affected with violent acute rheumatism; and we are inclined to think that he does not over-rate the proportion. The affection may exist in different degrees of intensity, and, *when the symptoms are not very violent,* it is not unfrequently overlooked by *cursory* observers. If, indeed, we were not afraid of startling our readers, we would go much farther than even M. B. has done, and say that, in *every* case of acute rheumatism, there exists a greater or less degree of inflammation, or—if that term be too obnoxious—of irritation in either the covering, or the lining membrane of the heart, or in both together. In proof of this statement, we would appeal to the pulse in rheumatism, which we have always found to be different from what it is in any other disease. This is new ground, and we are not without our fears of being charged with fancying distinctions, too fine-spun to be appreciable to other men's senses.* But we would willingly undertake, by the examination of a single case of acute rheumatism, to convince any physician of ordinary tact and power of observation, that the peculiarity of pulse to which we here refer is a thing of real existence, and not an imaginary creation of our own. We have repeatedly, in hospital, directed to it the attention of our medical friends, and we do not remember a single instance in which any of them failed to recognize it. It is much more easily distinguished than described, but the epithet *fleeting* is, in our opinion, the most appropriate which can be given to it. It throbs against the finger strong and full as in other inflammatory affections, but instead of continuing its impulse for an appreciable time, it flies onwards with the rapidity, as it were, of lightning. It differs from the rapid pulse produced simply by excessive loss of blood, in the circumstance of its being *more fleeting*, and in the occurrence of a longer interval between the several pulsations. It is also, as might be expected, much firmer, and much less compressible. It invariably attends upon the developed stage of acute rheumatism, whether bleeding has been practised or not; and by it alone—without a single question being put to the patient—without the slightest examination of his joints—a practitioner who has attended to the symptom in former cases, may be able to specify with confidence the nature of the affection. It is not always accompanied by a bellows-sound of the heart, although this is of much more frequent occurrence than is generally supposed, and may, by the stethoscope, be very often discovered to exist, when no pain in the region of the heart is complained of, and when the respiration, unless noted carefully, is not observed to be unnaturally short and hurried. We have been led to this digression by our conviction, that the peculiar state of pulse on which we have so much insisted, being, as it is, constantly present in acute rheumatism, and arguing, as it does, a highly irritated state of the heart, corroborates strongly M. B.'s notion respecting the *original* and *independent* affection of this organ.

Let us now return to M. B.'s treatise, from which we hope to glean for our readers many useful as well as interesting observations. The work is divided into six chapters, of which the first treats of the "law of coincidence of *endocarditis* and of *pericarditis* with acute articular rheumatism." In the remaining five chapters are discussed the symptoms, progress, duration, termination, seat, pathology, causes, nature, and treatment of acute rheumatism. We shall confine our attention, in a great measure, to the questions contained in this latter group; for the subject of the first chapter, having already been discussed in our author's "Treatise on Diseases of the Heart," was considered by us in our *critique* of that work, contained, as we have mentioned above, in our last Number.

As an apology, if any be needed, both for M. B. and ourselves, we think the following quotation not altogether inappropriate.

"It would at first sight (says our author) appear, that no subject can well be more trite and more exhausted than the history of rheumatism in general, and of acute rheumatism in particular. Such, however, is far from being the case, and

* We must not be understood to go quite so far as our Reviewer.

I venture to hope that the researches which form the subject of this work will be found deficient neither in interest nor in novelty." 1.

We shall pass over M. B.'s description of the symptoms of acute rheumatism, inasmuch as it contains nothing particularly worthy of notice. The delineation of them drawn by the master hand of our countryman, Sydenham, is so true to nature, that, for our own part, we feel but little inclined to look upon any other.

The truth of the following remark will, we believe, be assented to by every physician of experience.

"In general, all other circumstances being equal, the fixedness and persistence of acute articular rheumatism are *inversely* proportionate to the number of articulations affected." 71.

Our author thinks that the rapidity with which rheumatism shifts from one joint to another, is greatly exaggerated.

"The pain may, it is true, readily disappear, with or without the establishment of pain in any other joint, but it is not always so with the effusion, which, however, constitutes the essential element of the disease. The pain is, indeed, but a kind of neuralgia, symptomatic of the articular affection, as the stitch in the side is of pleurisy; and in both cases it is contingent rather than essential, for the pleurisy may exist without pain, as may also rheumatism of the joints, which is, in fact, nothing else than *a pleurisy of the synovial membranes.*" 72.

The peculiar manner of expression above made use of by M. Bouillaud, may convey very forcibly his meaning, but it is, at the same time, far from being correct; and we can foresee many disadvantages which would result from a general indulgence in it. The term pleurisy carries with it so obviously the idea of the pleura, that no disease which is not seated in that membrane, can with any degree of propriety be denominated by it. The most striking analogies in the symptoms, progress, or causes, are not sufficient to warrant such an abuse of language. We insist upon the point the more, because M. B. is rather prone to such a deviation from correct diction. In his present work, there is copied from his "Treatise on Diseases of the Heart," a passage in which he says that "rheumatism is, so to speak, nothing else than a *pericarditis* of the joints."

The mobility or vagabondism, if we may so call it, of rheumatism, has been urged by several French writers, against the notion of its being an inflammatory disease; and it is evidently with a view of meeting this objection, that our author endeavours to establish for it a character for greater stability and persistence. He might, however, have chosen many much more satisfactory arguments, for we can testify that, in rheumatism, it is no very uncommon occurrence for several ounces of fluid to be removed from the knee-joint in one single night. M. B. would appear, indeed, to be aware of this fact, for he qualifies his denial of the rapid absorption of the fluid, by saying that it does not "*always*" take place. Further on he adopts a much better line of argument, insisting upon the nature of the symptoms—the pain, heat, redness, and swelling; upon the co-existence of *endocarditis* and of *pericarditis*; upon the formation of pus in the joints; upon its analogy with pleurisy, bronchitis, &c. in which, as in rheumatism, a moderate degree of inflammatory action gives rise to a thin, serous secretion, and may then speedily subside, while a more intense inflammation produces pus and is of much longer continuance. Rheumatism, moreover, owes its development to the same atmospherical conditions as the diseases above detailed; it yields, like them, to extensive venæsection, judiciously prescribed; and the blood drawn in it presents the same buffy coat, the same cupped, firm, and glutinous coagulum. Such M. B. asserts is the nature of the blood even when it is drawn by cupping, as he found, among other instances, in the case of a pale, exsanguined person who had laboured under chronic rheumatism for five or six years, but in whom the disease had, about a fortnight before, assumed a subacute character. These qualities of the blood have been insisted on by many authors both ancient and modern. M. B. refers to Sydenham, Stoll, and Roche, and we may further quote the authority of Baglivi, who goes so far as to assert that such a state of the blood is actually the cause of the disease.

"For there prevails," says he, "at Rome a species of rheumatism, or disease attended with pains wandering over the whole body, *which undoubtedly, owes its origin to an inflammatory diathesis of the blood*."*

The same doctrine, we recollect, is maintained by Browne in his *Elementa Medicinæ*, in which he states that, unless as a consequence of external injury, there never does exist such a thing as a purely local inflammation, the whole mass of circulating blood being invariably altered from its natural condition. Baglivi further mentions that a corresponding change in the blood exists also in pleurisy, and he argues that

"On the ground of this analogy alone, we may safely reason from the treatment of the one disease to that which would be proper in the other, so as in fact to find that the same remedies are applicable to both.† Sir John Floyer would appear to have entertained the same opinion, for he alleges that 'pleurisy is nothing else than a kind of rheumatism.‡'" 21.

Our author scouts the idea of rheumatism being an *essential* fever, and he meets the objection of those who urge that the febrile symptoms sometimes continue after all affection of the joints has subsided, by insisting on the important fact that in such cases the heart or its capsule is invariably affected.

"The symptoms of such affection are so well marked, that they may be detected by any, even the least experienced, eye; but some men," M. B. adds, "are not less clever in finding out what does not exist (the *essentiality*, namely, of rheumatic fever) than they are apt to pass unnoticed that which is not only real but obvious." p. 13.

If the inflammatory nature of articular rheumatism be further questioned on the ground that its symptoms and ordinary termination differ from those of traumatic inflammation of the joints, M. B. would remind the objectors, that sufficient reasons for the differences may be found in the wider diffusion of rheumatism, which, attacking at once several joints, cannot, of necessity, affect any individual one so intensely as, if strictly confined to that one, it would most probably do; also, in the peculiar state of the blood above referred to which, indeed, may be the cause of such wider diffusion. This view of the subject M. B. confirms by a positive fact.

"When rheumatism, becoming chronic, is *fixed* in a joint for several months, it does actually produce the same lesions which simple and undoubted inflammation occasions. The *white-swelling* of rheumatic joints presents on dissection

* Opera Omnia Medico-Practica, &c. tom. ii. p. 157. Lipsiæ, 1828.

† Loc. citat. ‡ History of Cold Bathing, p. 10.

anatomical characters precisely similar to those which characterize white-swelling when the consequence of a wound." 109.

The above argument is not altogether free from fallacy. Dr. Barlow, in his able article on rheumatism in the *Cyclopædia of Medicine*, has shown that, after the subsidence in a joint of the specific action of rheumatism, there may be set up in it one of inflammation, which shall require to be treated as such, and which, if not controlled, may lead to the ordinary results—suppuration, ulceration, &c. In this manner are we inclined to account, in part, for the circumstance of rheumatism having been so often reported, by authorities of unquestionable veracity, to have terminated in the formation of pus. But at the same time it is a notorious fact that many of the cases in which such a termination is recorded, had really nothing whatever of rheumatism in their nature. In proof of this remark we would refer to a case related by Morgagni in his 57th Epistle, which, although styled rheumatism by Dr. Good, does not seem to have been considered by the author himself as such; to one related by Tissot in his "*Avis au Peuple sur sa Santé*," Cap. xi.; also to a case copied into the third volume of the "London Medical Repository" from the "*Bibliotheque Medicale*," which is reported as rheumatism, and in which four pints of sanious pus collected over and between the left gastrocnemii muscles. After a perusal of the details of this case no one, we apprehend, can for an instant doubt that it was one not of rheumatism, but of typhoid fever complicated, as such fever often is, with the formation of an abscess.

These remarks we feel assured are not only justified, but called for, by the loose and careless manner of authors, and reporters of cases, in assigning the name of rheumatism to a great number of affections which are really very different in their nature from that disease. This error is particularly common among Continental writers; and with the French it has, we are inclined to think, been not a little promoted by the retention of the old generic appellation, *arthrite*, which, if it conveyed a distinct idea for each separate and different affection that is usually classed under it, would be the most expressive term in existence. But a common name need not, and very often does not, convey the idea of more than one common quality, and we may be accused of unfairness in objecting to *Monsieur Arthrite*, that he is, in this respect no better than his neighbours. We maintain, however, that he is not quite so good as most of them, for they in general communicate the notion of some common and important *quality*, while he points only to a common *seat*. If all our nosology were arranged upon this principle, it would be beautifully simple, indeed, but we question whether it would have any thing else to recommend it.

Yet we are not prepared to assert that true inflammatory rheumatism *never* ends in suppuration;—what we maintain is, that such a termination is infinitely less frequent than, from the statements contained in books and periodical journals, one might be led to imagine. That there is something specific in the nature of rheumatic inflammation we firmly believe, but notwithstanding this we deem it not impossible, that the same thing may happen to it that we know happens to simple pleurisy and peritonitis, in which the effusion is ordinarily serous, but may become puriform, provided the inflammatory action rise in intensity, or contine long. M. B. to prove that such is actually the case, quotes the authority of Stoll, of Chomel, (whose cases demonstrate the fact though he himself denies it), of Moreau, Piorry, Raciborski,

Cruveilheir, &c. and he cites two cases which fell under his own observation. Of one of these, as it is interesting, we shall give a brief detail.

The patient, a young woman, had laboured under very acute articular rheumatism, and had been in hospital a fortnight when she died of a pleurisy, the existence of which was not discovered until the body was opened.

"The left knee-joint was red and dry, and the condyles of the femur were eroded; there was no pus. The right knee-joint was filled with genuine pus, mixed with synovial fluid. A portion of the crural vein near the joint contained pus, mixed with a reddish sanious fluid. Throughout the rest of its extent, including branches as well as trunk, it was filled with coagulated blood, and in several points a certain quantity of pus was recognized. Its sides, especially towards the knee, were thickened. The internal membrane was of a violet colour. The femoral artery was healthy, but the crural nerve was redder, and apparently larger than natural." 83.

These morbid appearances suggest many important considerations to which, however, we have not time to attend.

The several facts brought forward by M. B. are sufficient, he thinks, to demonstrate that the true seat of articular rheumatism is the synovial membrane, and that the ligaments and parts exterior to the joints are only secondarily affected. This opinion, we think, is strengthened by, at least, one of the cases contained in Sir B. Brodie's valuable work, in which, after rheumatism, a quantity of bloody serum was found in the joints. As we have not a copy of the new edition at hand, we are unable to give the reference. The synovial membrane has also been found covered with coagulable lymph in consequence of an attack of rheumatism. In the second volume of the "Dublin Hospital Reports" there is given, by Mr. M'Dowel, an account of the dissection of a woman who had been attacked, three weeks after delivery, with acute rheumatism, during the progress of which, peritonitis occurred and carried off the patient. "In the knee-joints the entire of the synovial membrane internally was covered with lymph, closely adherent to it, and in its appearance precisely similar to that effused on the peritoneum. In each articulation there were about two ounces of serous fluid, also resembling that found in the abdomen; bands formed of lymph stretched across the articulation in several places. On raising the lymph from the membrane, the latter did not present the vascularity which was exhibited by the peritoneum. The cartilages and bones were sound. The surrounding fibrous structure was unaltered."

We have dwelt thus at length upon the pathology of rheumatism, because we conceive it to be a subject of great importance, and one which is yet far from being sufficiently investigated.

The average duration of acute articular rheumatism is generally stated at from forty to fifty days, but M. B. assures us that, if his peculiar plan of treatment be strictly followed, the disease will, in general, be completely subdued by the end of the second week, often at a much earlier period. That plan and its merits we shall describe in its author's own words.

"The genuine specific for acute articular rheumatism, its *cinchona*, if I may so speak, is the antiphlogistic treatment, and above all, bloodletting, which is the prince of antiphlogistics." 123.

But in order that bloodletting shall produce all the effect of which it is capable, not only must it be sufficient in quantity, but the intervals at which it is repeated must be properly timed. Nor is it unimportant whether the abstraction of blood be local or general, or whether both of these modes be combined. From defective attention to these very necessary particulars bloodletting has not hitherto been found so efficacious a measure in the treatment of acute rheumatism as it would otherwise have been. M. B. proposes, therefore, the following plan, or, as he calls it, *formula* for the abstraction of blood, which, he says, is essentially the same as that formerly recommended by him in pneumonia, as well as in pericarditis.

"The patient, on the evening of his admission into hospital (provided he be strong, and have a good constitution), is bled to 16 ounces. From very plethoric patients 20 to 24 ounces are sometimes taken; but 16 ounces is the common *dose*.

Second day.—Two bleedings from the arm of from 12 to 16 ounces each, and in the interval between them, leeching or cupping (the latter by preference) to the extent of 12, 16, or 20 ounces. The cupping-glasses are applied around the affected joints, or to the præcordial region, if, as generally happens, the heart is implicated.

Third day.—One bleeding from the arm, and the abstraction of 12 to 16 ounces of blood by cupping from the joints, or from the region of the heart.

Fourth day.—The fever, pains, swelling, &c. have sometimes completely subsided by this day, in which case the further abstraction of blood is not practised; but should they still exist, the patient is once more to be bled to the extent of 12 or 16 ounces.

Fifth day.—Generally speaking the disease, by the fifth day, is in full progress towards resolution. Sometimes, however, the fever may still continue well marked, and in that case venesection to 12 ounces, or cupping to the extent formerly stated, becomes advisable.

From the sixth, seventh, or eighth day, the patient convalesces rapidly, and he may now begin to take food.

Should a relapse take place (which will sometimes happen, though not perhaps so often as after the common methods of treatment), bleeding may be again necessary. Thus, in a case in which the disease had been cut short by four bleedings, the patient relapsed, and before recovery took place, it was found necessary to bleed him again five times.

Slight relapses may be treated by emollients, abstinence, baths, anodynes, &c. the disease wearing itself out in a few days.

To escape relapses it is of paramount importance that patients guard most carefully against the slightest breath of cold." 135.

We cannot represent in too strong language the necessity of attending to this caution. We have known many lives lost, both in public and in private practice, by patients lying or sitting in the line of a draught of air from some contiguous door or window. There is no other cause which has any thing like so much influence in concentrating the disease upon the heart, or in rekindling it there, when, by proper treatment, it has been nearly extinguished. For a long time past we have been in the habit of ordering every rheumatic patient who has shown the slightest symptoms of heart affection, to have flannel immediately put on next his skin, and to be kept, as much as possible, in a uniform well regulated temperature. The amount of evil which we have avoided by this simple precaution, if it could be calculated, would, we feel assured, appear incredible.

The only auxiliaries to the plan of treatment above detailed which M. B. employs, are abstinence, emollient drinks, blisters, the application of compresses smeared with mercurial cerate, and so arranged as best to favor resolution, emollient cataplasms, baths, opium in moderate doses, and exhibited either internally or by the endermic method.

Sydenham has given a formula for bleeding in rheumatism, and so also has Roche, but neither of these, M. B. asserts, is active enough, nor do the bleedings prescribed by them succeed each other at sufficiently short intervals. Herein, indeed, rather than in quantity, consists the difference between our author's peculiar plan, and those of all other physicians.

Eighty-four cases of rheumatism treated by our author from the month of September, 1831,* till the same month in 1835, have been published in his clinical reports contained in the *Journal Hebdomadaire*. They all terminated favorably, with the exception of a single case, which occurred before he began to practise his peculiar method of bloodletting. Of 28 of these cases we have in the work before us either a detailed or a tabular report, from which it appears that the mean duration of the disease was nineteen days nearly, (the mean time of treatment was considerably less than this, but there are not sufficient data to calculate it with accuracy); the mean number of bloodlettings $3\frac{1}{2}$; the mean quantity of blood by venesection alone $45\frac{1}{2}$ ounces; the mean number of leeches applied, 26 nearly. In only four out of the 28 cases does cupping appear to have been practised, but during the last two years, M. B. has more frequently had recourse to that measure, preferring it to leeching. M. B. says that, although on an average rheumatism will be cured, as pneumonia also is, by the loss of from four to five pounds of blood, yet that, now and then, it may be necessary to withdraw six, seven, or eight pounds. In one of the cases above referred to, the patient lost ninety-six ounces. In one or two cases general bloodletting was not practised, the symptoms, which were not very severe, giving way to the application of leeches alone, or of leeches together with cupping-glasses.

We shall now, to give a clearer idea of M. B.'s method of treatment, copy, in a condensed form, the details of a single case.

The patient, a gunner, aged 28 years, nine or ten days after a fatiguing journey, during which he had been exposed to wind and rain, was attacked with rheumatism; and, on the 19th of December (five or six days later), he was admitted into the clinical wards. Previously to this, no treatment had been instituted.

20th. Joints of lower extremities, particularly knees, violently affected; those of upper extremities very slightly so. Trifling swelling of knee-joints, but no redness; motion intolerable; countenance flushed and animated; pulse 96 to 100, strong and full; skin covered with a clammy disagreeable perspiration; sleeplessness, thirst, loss of appetite; tongue white in centre, red at tip and along margins. Ordered to be bled to 16 ozs., the operation to be repeated during the day and to the same extent; infusion of elder-flowers and wild poppy, cataplasms, lavements, abstinence.

21st. Pains of lower extremities greatly relieved—those of upper aggravated; wrists and hands violently affected, elbows and shoulders less severely so. Venæsection to 16 ozs.—12 leeches to each hand. Contin. cætera.

22d. General relief—to be again bled.

* Some of these cases are referred to in our Number for April, 1835.

23d. Patient free from pain, and comfortable; fever has disappeared; pulse 80. Venæsection conditionally.

At 8 o'clock in the evening, pains of wrists and shoulders recurred; but no blood was drawn, and by morning they had subsided.

24th. Pulse still full, strong, and hard, 80 to 84 in the minute. To give the disease a finishing blow, venæsection to 12 ozs. was again ordered. Chicken broth permitted.

25th. Patient quite well. Diet increased.

No relapse took place, and on the 5th January the patient went out completely cured.

In this case, venæsection was practised five times, and the blood drawn was invariably covered with a greyish-white, firm, buffy coat. With each bleeding the proportion of serum increased. The same thing usually occurred in other cases.

M. B. refers to two cases, in which his peculiar plan of treatment was said to have been tried, without producing the decisive effects attributed to it by its author; but he subjoins reports of them which testify plainly, that although in each a large quantity of blood was drawn, yet that the intervals between each separate abstraction of it were greatly too long. He insists that, to give his method of cure a fair trial, it is necessary to enforce it faithfully and rigorously. Let but this be done, and he appeals with confidence to the result. Now such a condition, it must be granted, is nothing more than reasonable; and as the question is one of great importance, we should rejoice to see it brought to the test of experiment. Few, however, we fear, will be found bold enough to undertake the task, and still fewer to execute it; for there have, of late years, prevailed strong prejudices against bleeding largely in rheumatism. These prejudices may be traced, we think, to the recorded opinions of some of our standing authorities in medicine. Drs. Cullen, Fordyce, Heberden, Haygarth, Joseph Brown, and many others, concur in representing profuse bloodletting in rheumatism as not only inefficacious, but even detrimental. Haygarth and Brown state in positive terms, that it favors, and has often occasioned, fatal metastasis to the heart. There is, indeed, in the work before us an indirect admission, upon which those who find fault with M. B's practice are very likely to lay a fast hold. M. B. avows that it was not until after he had adopted his peculiar system of bleeding largely and at short intervals, that he discovered *endocarditis* and *pericarditis* to be the ordinary concomitants of acute articular rheumatism. This statement, we own, staggered us a little, although it has for a long time past been our conviction—a conviction founded on no inconsiderable experience—that the danger of bleeding largely in acute rheumatism has been, to say the least, greatly exaggerated. We have seen, at any rate, *pericarditis* display itself in numerous cases of rheumatism, in which not an ounce of blood had been drawn. Our opinion, therefore, is far from being unfavourable to M. B.'s plan of treatment, which merits, we think, an impartial trial. The question, we repeat, is a highly important one, and, like all other questions in practical medicine, can be decided only by oft-repeated observations, carefully made and faithfully reported.*

---

* The Reviewer, who is a talented hospital physician, entertains, in the view of the senior Editor, too favourable an opinion of M. Bouillaud's plan of treatment in acute rheumatism. This favourable opinion is, therefore to be considered as on the authority of the individual Reviewer only.—J. J.

